# Preventive Effect of Pine Bark Extract (Flavangenol) on Metabolic Disease in Western Diet-Loaded Tsumura Suzuki Obese Diabetes Mice

**DOI:** 10.1093/ecam/nep231

**Published:** 2011-03-17

**Authors:** Tsutomu Shimada, Mitsutaka Kosugi, Daisuke Tokuhara, Masahito Tsubata, Tomoyasu Kamiya, Mayu Sameshima, Rika Nagamine, Kinya Takagaki, Ken-ichi Miyamoto, Masaki Aburada

**Affiliations:** ^1^Research Institute of Pharmaceutical Sciences, Musashino University, Shinmachi Nishitokyo-shi, Tokyo 202-8585, Japan; ^2^Department of clinical pharmacy, Graduate School of Natural Science and Technology, Kanazawa University, Kakuma-machi Kanazawa-shi, Ishikawa, Japan; ^3^Tomei Atsugi Hospital, Atsugi City, Kanagawa, Japan; ^4^Toyo Shinyaku Co. Ltd, Tosu-Shi, Saga, Japan

## Abstract

It is known that the metabolic syndrome has a multi-factorial basis involving both genetic and environmental risk factors. In this study, Tsumura Suzuki Obese Diabetes (TSOD) mice, a mouse model of multi-factorial, hereditary, obese type II diabetes, were given a Western diet (WTD) as an environmental factor to prepare a disease model (TSOD-WTD) and to investigate the preventive effects of Pine bark extract (Flavangenol) against obesity and various features of metabolic disease appearing in this animal model. In contrast to control Tsumura Suzuki Non-obesity (TSNO) mice, TSOD mice were obese and suffered from other metabolic complications. WTD-fed TSOD mice developed additional features such as hyperinsulinemia, abnormal glucose/lipid metabolism and fatty liver. The treatment with Flavangenol had a suppressive effect on increase in body weight and accumulation of visceral and subcutaneous fat, and also showed preventive effects on symptoms related to insulin resistance, abnormal glucose/lipid metabolism and hypertension. Flavangenol also increased the plasma concentration of adiponectin and decreased the plasma concentration of TNF-**α**. We next investigated the effect of Flavangenol on absorption of meal-derived lipids. Flavangenol suppressed absorption of neutral fat in an olive-oil-loading test (*in vivo*) and showed an inhibitory effect on pancreatic lipase (*in vitro*). The above results suggest that Flavangenol has a preventive effect on severe metabolic disease due to multiple causes that involve both genetic and environmental risk factors. The mechanism of action might involve a partial suppressive effect of meal-derived lipids on absorption.

## 1. Introduction

Obesity, especially accumulation of visceral fat, is associated with a potential for harm and provides a pathological basis for various metabolic diseases, the development of diabetes/abnormal lipid metabolism/hypertension and other metabolic complications starting with insulin resistance and progressing to arteriosclerosis and ischemic cerebral/cardiac diseases. Prioritized exploratory research has sought materials for developing drugs to prevent and treat these disorders.

Pine bark extract (Flavangenol), obtained from the pine trees that grow on the west coast of France (*Pinus maritima*), contains oligomeric proanthocyanidin complexes (catechin oligomers) as the major ingredient. Pine bark extract is reported to be an antioxidant [1] and anti-inflammant [2], with the capacity to improve diabetic microvascular damage [3], vascular endothelial cell function [4–6] and ischemia/reperfusion-induced renal injury [7]. Clinical studies indicate that the extract is effective in the treatment of chronic venous insufficiency and retinal micro-hemorrhages [8].

The primary causes of obesity and metabolic diseases based on obesity include genetic factors [9–11] and environmental factors (overeating, stress, development of means of transportation). We previously developed an animal model of the disease, Tsumura Suzuki Obese Diabetes (TSOD), which spontaneously develops various features similar to those of human metabolic diseases, and confirmed that this is a spontaneous obese mouse model of type II diabetes [12–14]. In addition, Izumi et al. analyzed TSOD mice by the QTL mapping method and reported that this is a multi-factorial hereditary obesity/metabolic disease model with mutations in the gene loci controlling body weight, insulin level, fat level and adipocyte size [15, 16].

In this study, TSOD mice with genes for metabolic disease were given a Western diet (WTD) as an environmental factor, and the preventive effects of Flavangenol on various metabolic disease features were investigated. Furthermore, to clarify the mechanism of action of Flavangenol, we investigated the effect of Flavangenol on absorption of meal-derived lipids.

## 2. Methods

### 2.1. Experimental Materials

Flavangenol used in this study was supplied by Toyo Shinyaku Co. Ltd (Saga Prefecture), and contains 72.5% polyphenol (determined by the Folin-Denis method) including 5% proanthocyanidin B1, 2.98% catechin and 0.23% epicatechin.

The WTD, F2WDT (Oriental Yeast Co., Ltd, Tokyo), contains 19.82% casein, 0.3% l-cystine, 3.7458% cornstarch, 1.25%  *α*-Cornstarch, 34% sucrose, 1.0% soybean oil, 5.0% cellulose powder, 1.0% AIN-93 vitamin mixture, 3.5 AIN-93G mineral mixture, 0.25% choline bitratrate, 0.0042% t-butylhydroquinone, 20.0% milk fat (butter, etc.), 9.98% maltodextrin and 0.15% cholesterol. The total calories of nutritional elements were 450.8 kcal/100 g, and the calorie percentages were 17.8% for protein, 20.0% for lipid and 49.0% for carbohydrate. As a control diet, MF (Oriental Yeast Co. Ltd) was used as an “ordinary diet" (total calorie of nutritional elements: 360 kcal/100 g, calorie percentages: 23.6% for protein, 5.3% for lipid and 6.1% for carbohydrate).

### 2.2. Experimental Animals

TSOD mice and the corresponding control Tsumura Suzuki Non-Obesity (TSNO) mice (derived from the same ancestry but developing no metabolic disease), were purchased from the Institute for Animal Reproduction (Ibaraki Prefecture) at the age of 3 weeks. On the other hand, 5-week-old ddY mice were purchased from Tokyo Laboratory Animals Science Co. Ltd (Tokyo). The mice were acclimated for one week to the temperature of 23 ± 2°C and humidity of 55 ± 10%, and were given an ordinary MF powder diet and purified water *ad libitum*. After the acclimation, TSOD and TSNO mice were weighed and assigned to five groups (the TSNO-MF group: TSNO mice given MF; the TSOD-MF group: TSOD mice given MF; the TSOD-WTD group: TSOD mice given WTD; the TSOD-WTD-Flavangenol 3% group: TSOD mice given the WTD containing 3% Flavangenol; and the TSOD-WTD-Flavangenol 5% group: TSOD mice given the WTD containing 5% Flavangenol), ensuring that the body weight was distributed uniformly. Flavangenol was mixed well with the WTD so that the content was uniformly 3 or 5%. During the 8-week experiment, each group was given water and each defined diet *ad libitum*. All the animal experiment procedures were performed pursuant to the Animal Experiment Ethical Rules of Musashino University.

### 2.3. Components Examined During the Animal Experiment

All the animals were weighed once a week, and the food intake was determined every other week [17–20]. For investigating the time-course of changes in amounts of visceral and subcutaneous fat, the amounts of visceral and subcutaneous fat were determined at the start of the experiment and at following 4 and 8 weeks, a total of three times, by X-ray computed tomography (CT; Latheta, Aloka Co. Ltd. Tokyo) with scanning from the ensiform process to the sacral bone at distance intervals of 1.5 mm under anesthetization with Nembutal (50 mg/kg i.p.).

### 2.4. Glucose-Loading Test and Blood Pressure Determination

The glucose-loading test and blood pressure determination were performed 8 weeks after the start of the experiment. In the glucose-loading test, each mouse after fasting for one night was given oral glucose (2 g/kg); blood sampling from the orbital venous plexus was performed at the defined time points under non-anesthetized conditions. The plasma samples obtained by centrifugation were stored at −80°C until determination of glucose concentrations.

The blood pressure was determined with a non-invasive blood pressure meter (Softron Co. Ltd, Tokyo) by fixing each mouse in a fixing apparatus (Softron Co. Ltd) under non-anesthetized conditions. The cuff was set at the tail root to determine the systolic/diastolic/mean blood pressures.

### 2.5. Components Examined at the End of the Experiment

Under non-fasting conditions, each mouse was anesthetized with ether and the blood was drawn from the abdominal vena cava. The plasma samples obtained by centrifugation were stored at −80°C until blood biochemical tests could be performed. At the time of autopsy, the liver, mesenteric fat, perinephric fat, epididymal fat and retroperitoneal fat were isolated and weighed. Using the plasma samples obtained, the glucose, total cholesterol, triacylglycerol and free fatty acid levels were determined with biochemical tests kits obtained from Wako Pure Chemical Industries Ltd (Tokyo). The insulin level was determined with Rebis Insulin-Mouse-T (Shibayagi Co. Ltd, Gunma Prefecture), the adiponectin level with Mouse Adiponectin/Acrp30 Immunoassay (R&D Systems) and the TNF-*α* level with TNF-*α* ELISA (Bio Cosmo Co. Ltd, Tokyo).

### 2.6. Olive-Oil-Loading Test and Pancreatic Lipase Inhibition Test

The olive-oil-loading test was performed according to the method of Ninomiya et al. [21]. After fasting for one night, 6-week-old ddY mice were orally administered with Flavangenol (0.5 or 1 g/kg), and 30 min later, with olive oil (5 mL/kg). Blood sampling from the orbital venous plexus was performed at the defined time points under non-anesthetized conditions. The plasma samples obtained by centrifugation were stored at −80°C until determination of triacylglycerol concentrations.

The pancreatic lipase inhibition test was performed according to the method of Ninomiya et al. [21], using a general kit (Lipase Kit S: Dainippon Sumitomo Pharma Co. Ltd, Osaka) and porcine pancreatic lipase (L3126 Type II, Sigma-Aldrich, St Louis, MO).

### 2.7. Statistical Analysis

The data were displayed as mean ± standard deviation (SD). In each experiment, the inter-group difference was tested for significance by Dunnett's multiple comparison procedure with a significance level of 0.05.

## 3. Results

### 3.1. Changes in Body Weight and Food Intake

**Figure 1:**
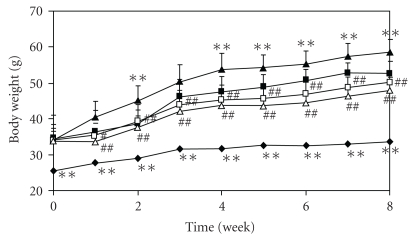
The effect of Flavangenol on body weight in mice. Closed diamond: TSNO-MF; closed square: TSOD-MF; closed triangle: TSOD-WTD; open square: TSOD-WTD-Flavangenol 3%; open triangle: TSOD-WTD-Flavangenol 5%. Data represent the mean ± SD of 8-9 animals. **P < .01, significant differences from the TSOD-MF group. ^##^ *P* < .05 and .01, significant differences from the TSOD-WTD group.

The body weight in the TSOD-MF group was significantly higher than in TSNO-MF group as the control group, and it became even greater in the TSOD-WTD group fed with the WTD. In the TSOD-WTD-Flavangenol groups, a dose-dependent and significant suppressant effect was seen on body weight from the first week after the start of the experiment, as compared with the TSOD-WTD group, and body weight lower than in the TSOD-MF group was seen from 4 weeks after the start of the experiment (Figure 1).  Table 1 shows the food and energy intake in each group at 8 weeks after the start of the experiment. The food and energy intake in the TSOD-MF group were significantly higher than in the TSNO-MF group, showing that TSOD mice overeat and ingest excessive energy. The food intake was significantly lower in the TSOD-WTD group than in the TSOD-MF group but the energy intake was not different. On the other hand, the food and energy intake were not different between the TSOD-WTD group and the TSOD-WTD-Flavangenol groups. The data on food and energy intake obtained at 2, 4 and 6 weeks after the start of the experiment in each group were almost the same as the data obtained after 8 weeks. 

### 3.2. Accumulation of Visceral and Subcutaneous Fat

**Figure 2:**
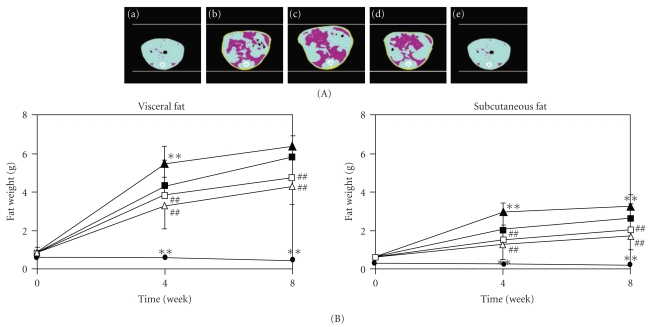
The effect of Flavangenol on the accumulation of adipose tissue in mice. (A) X-ray CT images of subcutaneous and visceral fat at 12 weeks. The yellow color shows the subcutaneous fat and the deep pink color shows the visceral fat. (a)TSNO-MF; (b)TSOD-MF; (c) TSOD-WTD; (d) TSOD-WTD-Flavangenol 3%; (e): TSOD-WTD-Flavangenol 5%. (B) Time-course of subcutaneous and visceral fat accumulation. closed diamond: TSNO-MF; closed square: TSOD-MF; closed triangle: TSOD-WTD; open square: TSOD-WTD-Flavangenol 3%; open triangle: TSOD-WTD-Flavangenol 5%. Data represent the mean ± SD of 8-9 animals. **P < .01, significant differences from the TSOD-MF group; ^##^ *P* < .01, significant differences from the TSOD-WTD group.

Figure 2(a) is a CT image from around the sixth lumbar vertebra of a representative mouse showing the average body weight of each group determined 8 weeks after the start of the experiment. The abdominal circumference increased more in the TSOD-MF group than in the TSNO-MF group and further increased markedly in the TSOD-WTD group. On the other hand, in groups treated with Flavangenol, the extreme increase in abdominal circumference seen in the TSOD-WTD group was prevented. The amount of visceral and subcutaneous fat was significantly higher in the TSOD-MF group than in the TSNO-MF group and further markedly higher in the TSOD-WTD group fed with WTD (Figure 2(b)). Both the accumulated visceral and subcutaneous fat amounts showed significantly higher values from 4 weeks after the start of the experiment. On the other hand, in the TSOD-WTD-Flavangenol groups, a significant suppressive effect on accumulation of both visceral and subcutaneous fat was seen from 4 weeks after the start of the experiment in comparison with the TSOD-WTD group, and the amounts of visceral and subcutaneous fat in the TSOD-WTD-Flavangenol groups became lower than in the TSOD-MF group. 

### 3.3. Tissue Weights and Autopsy

**Figure 3:**
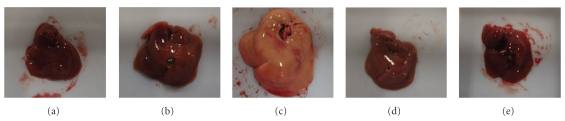
The effect of Flavangenol on the liver texture. (a) TSNO-MF; (b) TSOD-MF; (c) TSOD-WTD; (d) TSOD-WTD-Flavangenol 3%; (e) TSOD-WTD-Flavangenol 5%.

On comparing the TSNO-MF and the TSOD-MF groups, no significant difference was seen in liver weight, and no particular differences were seen in liver autopsy findings either. Between the TSOD-MF and the TSOD-WTD groups, a significant increase in liver weight due to the WTD was confirmed, and the autopsy findings also confirmed the marked liver enlargement and fatty liver. On the other hand, the liver enlargement and fatty liver seen in the TSOD-WTD group were markedly prevented by administration of Flavangenol (Figure 3 and Table 2). 

The epididymal and retroperitoneal fat accumulated about 5 and 3.5 times more, respectively, in the TSOD-MF group as compared with the TSNO-MF group, but there were no significant differences between the TSOD-MF and the TSOD-WTD groups. Mesenteric and perinephric fat also accumulated about 4.5 and 7 times more, respectively, in the TSOD-MF group as compared with the TSNO-MF group and accumulated further in the TSOD-WTD group. Administration of Flavangenol significantly suppressed fat accumulation in each visceral adipose tissue, and the amounts of mesenteric fat and perinephric fat were lower by 50 and 60%, respectively, in the TSOD-WTD-Flavangenol 5% group than in the TSOD-WTD group, and the amounts of epididymal fat and retroperitoneal fat were lower by 10 and 30%, respectively.

### 3.4. Biochemical Test Values

Table 3 shows the biochemical test values of the plasma samples obtained in each group 8 weeks after the start of the experiment. 

The non-fasting blood glucose level was not different between the TSNO-MF and TSOD-MF groups, but a significant increase was seen due to provision of the WTD in TSOD group. When Flavangenol was administered, the non-fasting blood glucose level was significantly lower in a dose-dependent manner as compared with the TSOD-WTD group. The fasting blood glucose level was significantly higher in the TSOD-MF group than in the TSNO-MF group and even higher in the TSOD-WTD group, but this increase was suppressed in the TSOD-WTD-Flavangenol groups in a dose-dependent manner. The total cholesterol level was significantly higher in the TSOD-MF group than in the TSNO-MF group and further higher in the TSOD-WTD group, but there were no significant differences between the TSOD-WTD group and TSOD-WTD-Flavangenol groups. The triacylglycerol level and free fatty acid level were significantly higher in the TSOD-MF group than in the TSNO-MF group, but no significant differences were seen between the TSOD-MF and the TSOD-WTD groups or TSOD-WTD-Flavangenol groups. The insulin level was significantly higher in the TSOD-MF group than in the TSNO-MF group and even higher in the TSOD-WTD group, but this increase was suppressed in the TSOD-WTD-Flavangenol groups in a dose-dependent manner. The adiponectin level was significantly lower in the TSOD-MF group than in the TSNO-MF group and still lower in the TSOD-WTD group, but this decrease was suppressed in the TSOD-WTD-Flavangenol groups in a dose-dependent manner. The TNF-*α* level was significantly higher in the TSOD-MF group than in the TSNO-MF group, but there were no significant differences between the TSOD-MF group and the TSOD-WTD group, and this increase was significantly suppressed in the TSOD-WTD-Flavangenol 5% group.

### 3.5. Glucose-Loading Test and Blood Pressure Determination

**Figure 4:**
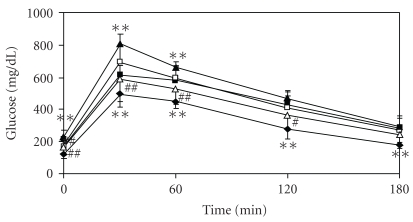
The effect of Flavangenol on the plasma glucose level in the oral glucose tolerance test. Closed diamond: TSNO-MF; closed square: TSOD-MF; closed triangle: TSOD-WTD; open square: TSOD-WTD-Flavangenol 3%; open triangle: TSOD-WTD-Flavangenol 5%. Data represent the mean ± SD of 8-9 animals. **P < .01, significant differences from the TSOD-MF group. ^##^ *P* < .01, significant differences from the TSOD-WTD group.

The blood glucose level was higher in the TSOD-MF group than in the TSNO-MF group at all times determined, and was even higher in the TSOD-WTD group than in the TSOD-MF group at 0.5 and 1 h after loading; postprandial hyperglycemia was seen in the TSOD-WTD group (Figure 4). Conversely, in the Flavangenol-treated groups, dose-dependent suppression was seen as compared with the TSOD-WTD group, and significant suppression was seen at 0.5, 1 and 2 h after loading in the TSOD-WTD-Flavangenol 5% group. 

**Figure 5:**
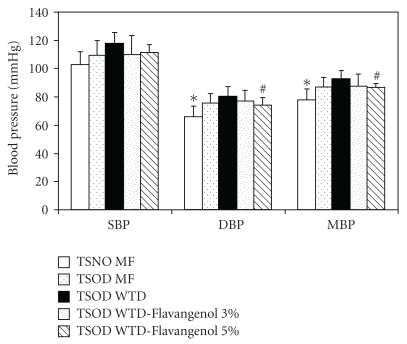
The effect of Flavangenol on blood pressure. Data represent the mean ±SD of 8-9 animals. *P < .05, significant differences from the TSOD-MF group; ^#^ *P* < .05, significant differences from the TSOD-WTD group.

The diastolic blood pressure and mean blood pressure were significantly higher in the TSOD-MF group than in the TSNO-MF group, and the hypertensive findings were confirmed in the TSOD-MF group (Figure 5). A comparison between the TSOD-MF and the TSOD-WTD groups revealed that the blood pressure tended to be higher in the latter. In spite of the increased blood pressure in the TSOD-WTD group, diastolic blood pressure and mean blood pressure were significantly lower in the TSOD-WTD-Flavangenol 5% group. 

### 3.6. Olive-Oil-Loading Test and Pancreatic Lipase Inhibition Test

**Figure 6:**
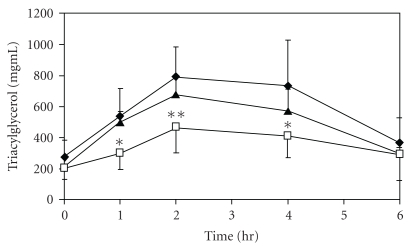
The effect of Flavangenol on plasma triacylglycerol elevation in olive-oil-loaded ddY mice Closed diamond: Normal (water); open triangle: Flavangenol 0.5 g/kg p.o.; open square: Flavangenol 1.0 g/kg p.o. Data represent the mean ±SD of six animals. *P < .05 and **P < .01, significant differences from the normal mice.

In the olive-oil-loading test, the plasma triacylglycerol level after loading with olive oil increased, showing a peak value at 2 h after loading, but this increase was inhibited by pretreatment with Flavangenol in a dose-dependent manner. Flavangenol (1.0 g/kg) administered orally significantly suppressed the plasma triacylglycerol level at 1, 2 and 4 h after administration (Figure 6). In addition, in the pancreatic lipase inhibition test, Flavangenol showed an inhibitory effect on pancreatic lipase activity, with an IC_50_ value of 335 *μ*g/mL. 

## 4. Discussion

Various metabolic diseases based on accumulation of visceral fat are known to become serious when some environmental factor is added [22, 23]. In this study, the preventive effect of Flavangenol on metabolic diseases was investigated by using a model that mimicked the modern human metabolic diseases by providing a WTD to TSOD mice, a spontaneous obese mouse model of type II diabetes. Additionally, for the purpose of clarifying the action mechanism of Flavangenol, we investigated the effect of Flavangenol on absorption of meal-derived lipids.

As reported previously, the TSOD-MF group showed increased food intake, obesity with predominant accumulation of visceral fat, insulin resistance and other metabolic complications, as compared with the TSNO-MF group [17–20]. When TSOD mice were given a WTD as an environmental factor, further significant obesity was seen from about 2 weeks after the start of the treatment, and significant accumulation of visceral fat (especially mesenteric fat and perinephric fat) and subcutaneous fat, marked liver enlargement and fatty liver were observed. On blood biochemical tests, significant increases of fasting/non-fasting blood glucose levels, total cholesterol and insulin levels and a significant decrease in adiponectin level were confirmed. It was clarified that more severe metabolic diseases appeared in the TSOD-WTD group following administration of a WTD as an environmental factor, as these mice have genes for metabolic disease. In the present study, the food intake was significantly lower in the TSOD-WTD group, but the converted energy intake was not lower.

The effect of Flavangenol added to the diet of the TSOD-WTD group was marked. Flavangenol did not influence the food and energy intake but, in a dose-dependent manner, suppressed the body weight increase due to a WTD. In the adipose tissues, which are closely related to an increase/decrease in body weight, it was clarified by X-ray CT analysis that Flavangenol showed a significant suppressive effect on accumulation of visceral and subcutaneous fat in a time-course manner. Looking at the suppressive effect on fat accumulation by visceral adipose tissue, a stronger effect was observed on mesenteric and perinephric fat, which increased more significantly on the WTD. When we previously investigated the effect of Flavangenol in a model developed by giving a high-fat diet to Sprague Dawley (SD) rats, we observed a significant suppressive effect on increase in body weight, accumulation of visceral fat and an increase in fecal excretion of total lipid in the group given a diet containing 2% Flavangenol (unpublished results). In the present study, similar results were obtained in the severe disease state that mimicked human disease by combining genetic and environmental factors. Additionally, it was also clarified that Flavangenol showed a preventive effect on liver enlargement and fatty liver. Recently, it has been strongly suggested that fatty liver is involved in onset and progression of non-alcoholic steatohepatitis (NASH) [24]. It is therefore anticipated that Flavangenol may also exert a preventive effect against NASH.

It was reported that visceral fat accumulation or adipocyte hypertrophy induces insulin resistance and the mechanism of action involves decreased secretion of adiponectin (which is known to improve arteriosclerosis and various metabolic diseases) and increased secretion of TNF*α* (which induces insulin resistance) [25]. In this study, Flavangenol significantly suppressed accumulation of visceral fat, significantly prevented the hyperglycemia and hyperinsulinemia observed in the TSOD-WTD group and prevented onset of abnormal glucose tolerance, suggesting a role in improved insulin resistance. In addition, since Flavangenol significantly increased the adiponectin level and significantly decreased the TNF-*α* level, it is suggested that the abnormal secretion of adipocytokines was normalized and onset of insulin resistance was prevented as a result, by the suppressive effect of Flavangenol on hypertrophy and accumulation of adipocytes. In this study, the body weight, amounts of visceral and subcutaneous fat and liver weight were lower in the TSOD-WTD-Flavangenol groups than in the TSOD-MF group. Furthermore, the plasma TNF*α* level was significantly lowered by Flavangenol, though not influenced by providing a WTD, suggesting that Flavangenol was effective not only for the obesity induced by a WTD but also on the original hereditary obesity of TSOD mice.

In this study, Flavangenol did not show a suppressive effect on the increases in blood levels of lipids, such as total cholesterol, triacylglycerol and free fatty acid levels, observed in the TSOD-WTD group. On the other hand, Flavangenol significantly suppressed accumulation of visceral and subcutaneous fat and onset of fatty liver. Mochizuki et al. [26] reported that Pine bark extract enhanced lipolysis by stimulation of *β*-receptors in a study using 3T3 cells. It is suggested that enhancement of lipolysis by Flavangenol through stimulation of *β*-receptors might be involved in the preventive effect of Flavangenol on onset of fatty liver and fat accumulation. Conversely, it is also suggested that a suppressive effect of Flavangenol on increased plasma triacylglycerol and free fatty acid levels was not seen, due to transfer to plasma of free fatty acids formed through lipid decomposition in the liver and adipocytes. It is known that fat accumulation in the liver and skeletal muscles or abnormal secretion of adipocytokines from hypertrophic adipocytes induces onset of insulin resistance. It is considered important that onset of fatty liver and fat accumulation was prevented by Flavangenol in this study, though Flavangenol did not show a preventive effect on increases in blood lipid levels.

Hypertension is one of the diagnostic criteria for the metabolic syndrome, and Flavangenol showed a significant antihypertensive effect (on diastolic and mean blood pressure). Pine bark extract was reported to show vascular protection due to an antioxidant effect [6], endothelium-dependent vasodilatation [27], improvement of endothelial cell function in hypertension patients [5] and activation of endothelial nitric oxide synthase (eNOS) in the endothelial cells [6]. Also, it is suggested that the suppressive effect of Flavangenol on hypertension seen in this study involves a direct effect on the endothelial cells and an indirect effect through suppression of the onset of insulin resistance.

**Figure 7:**
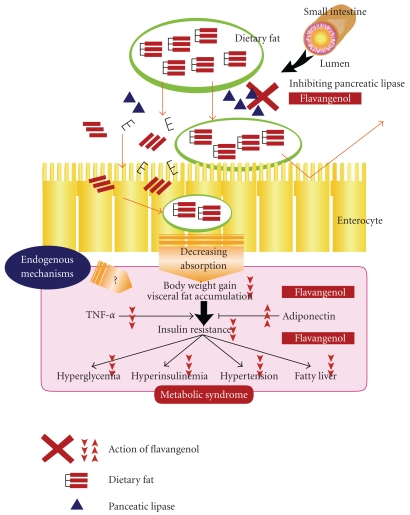
Mechanisms of action of anti-metabolic disease by Flavangenol.

In this study, more severe features of metabolic disease appeared in the TSOD-WTD group following administration of a WTD as an environmental factor to TSOD mice that had metabolic disease genes as a genetic factor. It was therefore suggested that the increased absorption of meal-derived lipids was involved in aggravation of various metabolic disease symptoms. It was reported that plant-derived polyphenols suppress absorption of triacylglycerol and cholesterol [28–30], suggesting that the effect of Flavangenol on prevention of metabolic disease observed in this study involves a lipid-absorption-inhibiting effect. In the *in vivo* experiment using ddY mice, Flavangenol showed a significant, dose-dependent suppressive effect on absorption of triacylglycerol. In addition, in the *in vitro* experiment, Flavangenol was confirmed to have an inhibitory effect on pancreatic lipase, which is the rate-determining enzyme involved in absorption of meal-derived triacylglycerol (IC_50_ value: 335 *μ*g mL^−1^). This effect was at the same level as the effects of tea catechins and heat-treated tea catechins, which are known to have an inhibitory effect on lipid absorption [31]. Apple-derived oligomeric procyanidins were reported to have a lipase-inhibiting effect and show an inhibitory effect on lipid absorption in mice and humans [32], supporting the results obtained in this study. The fact that Flavangenol increased fecal excretion of total lipid in the SD rats loaded with a high-fat diet (to be submitted) endorses the results obtained in this study. In addition, Pine bark extract was reported to inhibit also *α*-Glucosidase involved in absorption of meal-derived sugars [33]. It is thus suggested that the mechanisms of the hypoglycemic effect and metabolic-disease-preventing effect of Flavangenol observed in this study might have included the inhibitory effect on absorption of meal-derived lipids and sugars (Figure 7). However, inhibitory activity on the enzyme is unlikely the main underlying mechanism. 

Although our findings suggest beneficial metabolic effects of Flavangenol in a genetic animal model of type 2 diabetes and obesity, the endogenous mechanisms need to be further investigated in future.

## Funding

“High-Tech Research Center" Project for Private University: matching fund subsidy from “Ministry of Education Culture, Sports, Science and Technology" 2004–2008 of Japan.

## Figures and Tables

**Table 1:** The effects of Flavangenol on food intake and energy intake at 8 weeks treatment.

	TSNO MF	TSOD			
		MF	WTD	WTD-flavangenol 3%	WTD-flavangenol 5%
Food intake (g/day/body)	4.5 ± 0.3*	5.2 ± 0.3	4.3 ± 0.0**	4.0 ± 0.2	4.3 ± 0.4
Energy intake (kcal/day/body)	16.2 ± 1.1*	18.6 ± 0.9	19.6 ± 0.2	19.4 ± 1.9	19.2 ± 1.7

Data represent the mean ± SD of 8-9 animals.

*P < .05 and **P < .01 Significant differences from the TSOD-MF group.

**Table 2:** The effects of Flavangenol on liver and visceral fat weight for each viscus.

	TSNO-MF	TSOD			
		MF	WTD	WTD-flavangenol 3%	WTD-flavangenol 5%
Liver (g)	1.69 ± 0.19	1.89 ± 0.17	3.03 ± 0.64**	1.79 ± 0.11^##^	1.79 ± 0.10^##^
Epididymal fat (g)	0.59 ± 0.15*	2.92 ± 0.27	3.09 ± 0.23	3.11 ± 0.33	2.77 ± 0.29^#^
Mesenteric fat (g)	0.35 ± 0.I6**	1.60 ± 0.39	2.02 ± 0.28*	1.21 ± 0.27^##^	1.04 ± 0.36^##^
Retroperitoneal fat (g)	0.17 ± 0.05**	0.65 ± 0.09	0.78 ± 0.20	0.56 ± 0.05^##^	0.57 ± 0.07^##^
Perinephric fat (g)	0.08 ± 0.02**	0.59 ± 0.13	0.94 ± 0.13**	0.41 ± 0.11^##^	0.35 ± 0.12^##^

Data represent the mean ± SD of 8-9 animals. *P < .05 and **P < .01 Significant differences from the TSOD MF group. ^#^ *P* < .05 and ^##^ *P* < .01 Significant differences from the TSOD-WTD group.

**Table 3:** The effects of Flavangenol on biochemical parameters of plasma.

	TSNO-MF	TSOD			
		MF	WTD	WTD-flavangenol 3%	WTD-flavangenol 5%
Non-fasted glucose (mg/dL)	169.1 ± 20.7	172.0 ± 22.3	330.0 ± 126.7**	178.4 ± 32.8^##^	168.2 ± 17.0^##^
Fast glucose (mg/dL)	120.0 ± 23.8*	168.2 ± 35.6	228.4 ± 42.6**	172.8 ± 42.7^#^	163.2 ± 44.0^##^
Total cholesterol (mg/dL)	113.9 ± 12.4**	170.1 ± 27.7	260.1 ± 27.0**	255.9 ± 31.8	247.0 ± 21.6
Triacylglycerol (mg/dL)	118.9 ± 32.0**	198.3 ± 38.2	239.4 ± 52.2	231.7 ± 50.6	261.7 ± 59.4
Free fat acid (mEq/l)	1.17 ± 0.14**	1.46 ± 0.27	1.69 ± 0.27	1.68 ± 0.35	1.70 ± 0.16
Insulin (ng/mL)	2.8 ± 1.3	15.4 ± 14.3	143.0 ± 81.4**	36.2 ± 62.9^##^	10.3 ± 13.6^##^
Adiponectin (*μ*g/mL)	5.20 ± 0.71**	2.71 ± 0.51	1.75 ± 0.29**	2.23 ± 0.36^##^	2.41 ± 0.34^##^
TNF-*α* (pg/mL)	4.7 ± 0.3**	11.1 ± 3.1	10.4 ± 3.9	10.0 ± 2.1	7.2 ± 1.3^##^

Data represent the mean ± SD of 8-9 animals. *P < .05 and **P < .01 Significant differences from the TSOD-MF group. ^#^ *P<*.05 and ^##^ *P* < .01 Significant differences from the TSOD-WTD group.
